# Role of Flavonoids in Protecting Against Neurodegenerative Diseases—Possible Mechanisms of Action

**DOI:** 10.3390/ijms26104763

**Published:** 2025-05-16

**Authors:** Elżbieta Rębas

**Affiliations:** Department of Molecular Neurochemistry, Medical University of Lodz, 90-419 Lodz, Poland; elzbieta.rebas@umed.lodz.pl

**Keywords:** flavonoids, neuroprotection, anti-oxidative, anti-inflammatory action, Alzheimer’s disease, Parkinson’s disease

## Abstract

Neurodegenerative and mood disorders represent growing medical and social problems, many of which are produced by oxidative stress, neuroinflammation, disruption in the metabolism of various neurotransmitters, and some disturbances in lipid/carbohydrate homeostasis. Biologically active plant compounds, including flavonoids, have been shown to exert a positive impact on central nervous system function. This review assesses the studies of naturally occurring flavonoids belonging to various polyphenol subclasses and their mechanisms of neuroprotective action, especially against neurodegenerative disorders such as Alzheimer’s disease and Parkinson’s disease. Most of the studied phytochemicals possess anti-oxidative, anti-inflammatory, and neuroprotective properties. These phytochemicals have been considered as compounds that reduce the risk of developing Alzheimer’s and Parkinson’s diseases and can be used in the treatment of neurological diseases. The neuroprotective actions of some flavonoids may entail mechanisms that regulate reactive oxygen species generation and modify inflammatory pathways, and they should be considered as therapeutic agents.

## 1. Introduction

Alzheimer’s disease, Parkinson’s disease, stroke, and mood disorders pose serious medical and social problems. All of them are neurodegenerative disorders with cognitive deficit as a main symptom. It has been observed that an increasing number of patients suffer from Alzheimer’s disease and Parkinson’s disease each year. Oxidative stress, the disrupted metabolism of some neurotransmitters, disturbed calcium homeostasis, gene mutations, and neuroinflammation are considered the main factors responsible for the development of neurodegenerative diseases [1,2]. Neurodegenerative diseases affect the functions of glial cells, neurons, and immune cells in the central nervous system (CNS), leading to neuronal death and cognitive decline [1].

Parkinson’s disease (PD) is a complex, age-related, and the most common neurodegenerative movement disorder associated with dopamine deficiency and both motor and nonmotor deficits. Various environmental, cellular, and genetic factors increase PD risk, leading to mitochondrial dysfunction, oxidative stress, protein aggregation (e.g., of α-synuclein), impaired autophagy, impaired neurotransmitter metabolism (e.g., of dopamine in substantia nigra), and neuroinflammation. Recent studies, such as epidemiological studies, indicate that neuroinflammation is not only a response to the ongoing neurodegeneration but may be an essential upstream contributor to α-synuclein aggregation and neurodegeneration progression. Interestingly, peripheral inflammatory processes (e.g., type 2 diabetes, high cholesterol levels, and inflammatory bowel disease) elevate PD risk [2]. Parkinson’s disease treatment is mainly symptomatic and often accompanied by serious side effects. Therefore, naturally occurring agents, including flavonoids, which reduce oxidative stress and neuroinflammation without side effects, may be helpful in the protection and treatment of Parkinson’s disease [3].

The other neurodegenerative disease is Alzheimer’s disease (AD), which is the most common type of dementia. It is a clinical syndrome characterized by a progressive decline in cognitive functions like memory, language, personality, and behavior, which causes a loss of abilities required to perform basic activities of daily living. The main changes observed in the brain during Alzheimer’s disease involve the deposition of amyloid-β (Aβ), phosphorylated tau, and intraneuronal neurofibrillary tangles in the parenchyma and the cerebral vasculature and the gradual loss of synapses. It is still unclear what exactly drives the progression of the disease. Genetic mutations are important causes of AD, but environmental or lifestyle factors, such as inadequate diet, reduced physical activity, stress, and some diseases, such as diabetes, cerebrovascular disease, and head injury, can increase the risk of AD. Additionally, conditions such as vascular abnormalities, detrimental changes in mitochondria, oxidative stress, reduced brain glucose utilization, and neuroinflammation currently seem to be significant factors of AD progression. Promising pharmacological therapies include anti-amyloid-β, anti-tau, anti-oxidative, and anti-inflammatory strategies, but lifestyle-based prevention trials, e.g., healthy diet, resulted in cognitive improvement in people with an increased risk of dementia and the slower development of AD [3,4,5].

## 2. Flavonoids

Flavonoids are plant-derived polyphenols. Polyphenols, such as flavonoids, stilbenes, lignans, phenolic acids, tannins, and chalconoids, occur naturally in fruits, vegetables, honey, and some beverages, like tea and wine. Various plant parts are sources of dietary polyphenols, e.g., grains, barks, roots, stems, leaves, skins, and flowers. The color of flowers, fruits, leaves, and root vegetables depends on the presence of these plant pigments [6,7]. They exert various beneficial effects on health due to their anti-inflammatory, anti-microbial, anti-oxidant, antimutagenic, and anti-cancer properties. Additionally, they can modify the metabolism or action of many neurotransmitters [7,8]. Due to the very low toxicity of phytochemicals, they are considered a safer alternative to the currently used synthetic preparations for neurodegenerative disease treatment, which may exert many adverse side effects. The absorption of flavonoids is relatively low, but the systematic consumption of flavonoid-rich products may be enough to attain their protective levels in the body. In some cases, during the frequent use of high amounts of polyphenols as dietary supplements, there is the risk of negative effects on various tissues, e.g., the thyroid gland [8,9]. The ability of flavonoids to cross the blood–brain barrier (BBB) is crucial for their therapeutic effects in neurological diseases [10]. On the other hand, an excess of these active compounds in the brain, due to their rapid transport via the blood–brain barrier, can lead to some toxic effects, e.g., as seen with quercetin.

Flavonoids were reported to exert anti-neuroinflammatory effects via various signaling pathways (e.g., the inhibition of nuclear factor kappa-light-chain-enhancer of activated B cells (NF-κB) pathway, activation of anti-oxidative enzymes, and removal of reactive oxygen species (ROS)). In this review, we focus on the neuroprotective actions of selected flavonoids, including the modulation of the neuroinflammatory processes, the modification of neurotransmitter metabolism, anti-oxidative action linked to neurodegenerative diseases, and their potential applications as neuroprotective agents.

Flavonoids are the largest group of polyphenols and are divided into several subclasses according to their structures: flavones, flavonols, flavanones, flavanols, isoflavonoids, anthocyanins, and chalcones [10]. The characteristics of the flavonoids from different subclasses that are described in brief in this review are presented in Table 1.

As was mentioned above, the absorption of flavonoids is rather low and depends on the structural form of these compounds, which occur in plants. Only aglycones and some glucosides can be absorbed in the small intestine in their unchanged form. Also, the methylation of flavonoid aglycones upon ingestion may improve their bioavailability compared to the native glycosylated form. E.g., the native form of quercetin is poorly absorbed as rutin (quercetin-3-rutenoside). In the colon, quercetin is converted to an aglycone form with better bioavailability. After ingestion, flavonoids can undergo glucuronidation (e.g., isorhamnetin-3-glucuronide, quercetin-3-glucuronide, and quercetin-5′-glucuronide are formed from quercetin), sulfation (e.g., quercetin-3-sulfate), and glycosylation (e.g., quercetin-3-glucoside, quercetin-4′-glucoside) to different degrees of availability. Additionally, flavonoids can interact with other dietary components that reduce their absorption, e.g., milk proteins or fiber [11]. The very important role of gut microbiota is in their generation of various metabolites of flavonoids, sometimes more active and better absorbed than precursors. One good example may be the protocatechuic acid generated from various flavonoids, including quercetin and anthocyanins, which can pass the blood–brain barrier and have a proven role in the prevention of neurodegenerative processes responsible for Alzheimer’s disease and Parkinson’s disease development. Similarly, two metabolites of EGCG generated by the gut microbiome, 5-(40-hydroxyphenyl)--valerolactone-30-sulfate and 5-(40-hydroxyphenyl)--valerolactone-30-O-glucuronide, can reduce AD risk and possess anti-inflammatory properties. The other active in CNS metabolites of flavonoids with anti-inflammatory and neuroprotective actions are urolithins directly synthesized from ellagic acid. However, ellagic acid is a derivative obtained from complex heterocyclic ellagitannin compounds in the presence of gut microbiota [11].

Therapeutic properties of flavonoids are very significant for human health despite their poor bioavailability due to the wide spectrum of dietary products that contain flavonoids and their conversion to active metabolites with improved bioavailability by colonic bacteria. It is worth mentioning that more than one biologically active compound exists in many plant sources, e.g., herbal plants, citrus fruits, and grapefruit [11,12,13,14]. It was confirmed that natural standardized grape (*Vitis vinifera* L.) extract containing a phytocomplex of anthocyanins and proanthocyanidins (grape juice is characterized by the presence of anthocyanins, malvidin, cyanidin, and proanthocyanidins, in the form of monomers, oligomers, and polymers: catechins, epicatechins, procyanidin B1, and procyanidin B2) improved cognitive function in a healthy aging population. The results of the aforementioned study showed that 250 mg/day of Cognigrape^®^ supplementation improved cognition in healthy older men and women [12]. A similar phytocomplex was identified in Nasco pomace grape (*Vitis vinifera* L. ssp. vinifera)—gallic acid, catechin, epicatechin, procyanidin B2, and quercetin [13]. *Myrtus communis* L., a Mediterranean plant used, e.g., in liquor production, possesses anti-oxidant and anti-inflammatory properties, as confirmed in a study on the neuroprotective potential of Myrtus berry by-products in PC12 cells exposed to 6-hydroxydopamine (6-OHDA) used as a neurodegeneration PD model [14]. Phytochemicals such as gallic acid, hyperoside, ellagic acid, quercitrin, quercetin-3-O-galactoside, quercetin-3-O-rhamnoside, cyanidin 3-O-glucoside, petunidin 3-O-glucoside, peonidin 3-O-glucoside, malvidin 3-O-glucoside, petunidin-3-O-arabinoside, malvidin 3-O-arabinoside, gallotannins, and ellagitannins are found in myrtle berries [14]. Also, it has been evidenced that the *Ginkgo biloba* leaf extract phytocomplex improves cognitive functions in Alzheimer’s disease, stroke, Huntington’s disease, and Parkinson’s disease [15].

Flavonoids existing together in plant sources (e.g., fresh, juice, extract, infusion) exert similar properties and mechanisms of action, including anti-oxidative, anti-inflammatory, and neuroprotective properties. For example, hesperidin alone or in combination with other flavonoids (e.g., diosmetin) shows neuroprotective properties against AD, PD, and other disorders associated with cognitive decline. Herbal preparations containing phytocomplexes have been used in traditional medicine for centuries. There are some known traditional Chinese medicinal formulations used in AD or PD treatment with several flavonoid, alkaloid, and other polyphenol components. They contain a mixture of flavonoids such as phillyrin glycoside, quercetin, glycyrrhizic acid, luteolin, baicalin, baicalein, hesperidin, hesperitin, naringin, naringenin, mangiferin, and licochalcone A [11]. Currently, the beneficial effects of hesperidin, epigallocatechin gallate, anthocyanins, and quercetin in the treatment of neurodegenerative disorders have been confirmed in some pre-clinical and clinical trials [11].

## 3. The Role of Various Classes of Flavonoids in Neurodegenerative Protection

### 3.1. Flavones

#### 3.1.1. Chrysin (5,7-Dihydroxyflavone)

Chrysin is a flavone found mainly in honey, propolis, and passion flowers and is also an ingredient in dietary supplements. Like other flavonoids, chrysin has been reported to show various biological activities such as anti-inflammatory, anti-oxidative, anti-allergic, and anti-cancer effects [10]. It can protect against or inhibit PD development in different ways: anti-oxidative action (increasing the synthesis of anti-oxidative enzymes), the stimulation of apoptosis, and the modulation of dopamine metabolism. The inhibitory effect of chrysin on neuroinflammation was evidenced in various in vitro studies and in animal models. Chrysin has been reported to improve cognitive deficits and reduce brain damage induced by chronic cerebral hypoperfusion in rats. Furthermore, it can protect against focal cerebral ischemia/reperfusion injury in mice through the attenuation of inflammation [16]. The anti-neuroinflammatory effects of chrysin via the NF-κB signaling pathway are a common mechanism. NF-κB is involved in the pro-inflammatory pathway and is linked to tumor necrosis factor α (TNF-α), nitric oxide (NO), interleukin-β (IL-1β), interleukin-8 (IL-8), and ROS production by microglia in PD.

Studies on BV2 microglial cells (the widely used in vitro neurodegenerative disease model) and primary microglia cells have demonstrated that chrysin pretreatment protects cells against lipopolysaccharide (LPS)-induced activation and the increased production of NO, IL-6, and TNF-α in a dose-dependent manner. Chrysin can inhibit the gene expression of IL-6, TNF-α, and TNF receptor-associated factor 6 (TRAF6) as well as induce the expression of A20, an inhibitor of inflammation, both at the mRNA and protein levels in BV2 microglial cells and primary microglia cells. A20 is a central negative regulator of Toll-like receptors (TLRs) and the TNF-α receptor pathway. NF-κB activation in the presence of IL-1β, TNF-α, IL-6, and LPS is terminated by the ubiquitin-editing enzyme coded by A20. These results were consistent with the results from other in vivo experiments, and it was observed that chrysin could reduce the production of TNF-α and IL-6 caused by LPS and upregulate A20 expression in the mouse cerebral cortex in the presence and absence of LPS. This upregulation affects TRAF6 polyubiquitination, which, in turn, can modify the inhibitory effects of chrysin on the NF-κB pathway. Additionally, chrysin decreased serum TNF-α levels when compared to the group treated with LPS only. All these results indicate that chrysin exerted an anti-neuroinflammatory effect in both BV2 and primary microglial cells induced by LPS and in in vivo conditions [17].

The neurotoxin 6-hydroxydopamine (6-OHDA) is widely used to obtain the experimental model of Parkinson’s disease. Chrysin can inhibit the 6-OHDA-induced NF-κB phosphorylation and reduce its transcriptional activity in PC12 cells [18,19]. It can also prevent the increase in TNF-α and IL-1β levels triggered by 6-OHDA and attenuate the upregulation of NF-κB levels in the striatum of 6-OHDA-treated PD mouse models. Thus, it suppresses the production of pro-inflammatory cytokines, such as IL-1β, TNF-α, IL-6, and interferon γ (IFNγ) in the nigrostriatal neurons [19,20]. Similar effects have been observed in the SNpc (substantia nigra pars compacta) using the MPTP (1-methyl-4-phenyl-1,2,3,6-tetrahydropyridine)-treated mouse models of PD [19,21]. MPTP can pass through the BBB, and in the brain, it is converted to its toxic metabolite, methyl-4-phenylpyridinium (MPP^+^), which destroys the dopaminergic neurons in the substantia nigra of the brain. Chrysin has been shown to exert anti-inflammatory effects via the inactivation of TLR-4/NF-κB inflammatory pathway [19].

Besides the inhibition of pro-inflammatory cytokines, it has been confirmed that chrysin can upregulate anti-inflammatory cytokines like IL-4 and IL-10 [19,22].

Other mechanisms of anti-inflammatory action of chrysin comprise the inhibition of the expression of cyclooxygenase-2 (COX-2), myeloperoxidase (MPO), and nitric oxide synthase (iNOS), which are highly implicated in inflammatory responses [22]. Additionally, chrysin stimulates the expression of anti-oxidative enzymes such us catalase (CAT), superoxide dismutase (SOD), and phase II detoxication enzymes, e.g., heme oxygenase-1 (HO-1) [18]. Its other suggested mechanism of action involves the increased expression and transcriptional activity of Nrf2 (nuclear factor erythroid 2-like 2; NFE2L2). Nrf2 promotes the upregulation of the Nrf2/anti-oxidant response element (ARE) anti-oxidant defense pathway. In this way, chrysin may exert beneficial effects against oxidative damage, which is one of the key pathogenic mechanisms observed in PD [13,14]. Chrysin and protocatechuic acid used together increase the activity of CAT and SOD in 6-OHDA-treated PC12 cells [19].

Studies show that the drug class thiazolidinediones, used in type 2 diabetes treatment, inhibit dopaminergic neuron loss by peroxisome proliferator-activated receptor gamma (PPAR-γ) activation and act in a neuroprotective manner in PD animal models. Some authors speculate that chrysin, as a PPAR-γ agonist, also exerts neuroprotective action by PPAR-γ activation [18]. PPAR-γ is involved in fatty acid storage and glucose metabolism regulation.

Using the amyloid-β precursor protein (APP)/PS1 mouse model of Alzheimer’s disease, it was confirmed that chrysin reduced cognitive impairment in AD by decreasing the levels of amyloid-β and phosphorylated tau, along with the dual-inhibitory activity against β-site-amyloid precursor protein cleaving enzyme 1 (β-secretase 1, β-site APP cleaving enzyme 1, BACE1) and glycogen synthase kinase 3β (GSK3β) [23].

It is known that memory deficit in dementia and Alzheimer’s disease can be a result of decreased levels of thyroid hormones, i.e., hypothyroidism. It was demonstrated that a one-month treatment of chrysin improved memory in adult female mice C57BL/6 with hypothyroidism induced by the continuous exposure to methimazole (MTZ). The mechanism of chrysin action comprises the decline in the glutamate levels and the decreased Na^+^, K^+^-ATPase activity in both the prefrontal cortex and hippocampus in hypothyroid mice. Acetylcholine esterase activity was not altered by chrysin in either cerebral structure [24].

The above examples indicate that chrysin can exert a positive effect on neurodegenerative disease symptoms through various mechanisms. Chrysin has low bioavailability and is poorly absorbed when taken orally. Thus, this flavone should be consumed through one’s daily diet to reap the benefits of its protective properties. A dose of 0.5 to 3 g of chrysin per day is considered safe [25].

#### 3.1.2. Luteolin (3′,4′,5,7-Tetrahydroxyflavone) (Other Names: Luteolol, Digitoflavone, Flacitran, and Luteoline)

Another flavone is luteolin, which is found in some vegetables, including celery, carrots, parsley, broccoli, and artichoke, and in spices and herbs like green pepper, thyme, dandelion, perilla, chamomile tea, peppermint, rosemary, navel oranges, olive oil, ginkgo biloba, and oregano. Luteolin is an important natural polyphenol that shows anti-inflammatory, anti-oxidant, anti-cancer, cytoprotective, and macrophage polarization effects [10]. It possesses hepatoprotective, cardioprotective, and neuroprotective properties. Luteolin can suppress neuroinflammation through several mechanisms involving a decreased release of cytokines, such as NO, the depletion of reactive oxygen species, calcium influx, and the inhibition of the Akt and NF-κB pathway, and it is a potent free radical scavenger [26].

Using an animal model of Alzheimer’s disease, with triple-transgenic mice (3 × Tg-AD) (harboring PS1 (M146V), APP (Swe), and tau (P301L) transgenes), it was demonstrated that the level of inflammatory cytokines (TNF-α, IL-1β, IL-6, NO) and COX-2 and iNOS protein expression were significantly higher in 3 × Tg-AD mice compared with WT control mice. Luteolin reduced the level of these inflammatory cytokines and the expression of COX-2 and iNOS. Similar results were observed in the LPS-induced inflammation C6 cell model; thus, luteolin showed an inhibitory effect on neuroinflammation in both animal and cell models of AD. Luteolin reduced the expression of ER stress-related proteins such as GRP78 (78 kDa glucose-regulated protein or heat shock 70 kDa protein 5 (HSPA5, BIP)) and IRE1α (the serine/threonine protein kinase/endoribonuclease inositol-requiring enzyme 1-α) and the expression and co-localization of glial fibrillary acidic proteins (GFAPs) in AD animals and C6 cell models. Reactive astrocytes are characterized by increased GFAP expression. GFAP expression is significantly higher in the cerebral cortex of 3 × Tg-AD mice than that of control mice, indicating an increase in reactive astrocytes in this model. Aβ deposition is not crucial for glial cell activation in the early stage of AD, before Aβ deposition occurs. Luteolin treatment reduced the expression of GFAP in the cerebral cortex of 3 × Tg-AD mice, suggesting a suppressive role of this flavone in the overactivation of astrocytes in AD. It is known that ER stress activates NF-kB, and it has been shown that luteolin reduces the inflammatory signaling by decreasing the level of p-NF-κB (phospho-NF-κB) and p38 mitogen-activated protein kinase (38MAPK). Additionally, it was found that luteolin treatment dose-dependently improved spatial learning and memory impairment in 3 × Tg-AD mice using behavioral tests (e.g., the Morris water maze) [27].

The inhibition of inducible NO synthase, cyclooxygenase-2, TNF-α, and interleukin-1β, as well as a decrease in nitric oxide and prostaglandin E_2_ (PGE2) levels mediated by luteolin, was observed in other studies that used murine BV2 microglia and LPS-induced pro-inflammatory mediator production as the experimental model of neurodegeneration. Microglial activation is one of the factors for neuroinflammation, which results in brain damage during neurodegenerative diseases. The preincubation of microglia with luteolin reduced LPS-induced NF-κB activation; thus, this is suggested to be a mechanism of action [28].

The inhibitory effect of luteolin on interleukin-1β expression and ER stress was also confirmed in studies using the hippocampi of luteolin-injected APP23 mice, which express human APP751 complementary DNA with a Swedish double mutation on the C57BL/6 genetic background (an AD animal model that develops an extensive amyloid-β pathology). Animals untreated with tested flavone (DMSO-injected) represented a comparative group.

Additionally, the reduced level of cluster of differentiation 68 (CD68), a transmembrane protein, in the brain was observed after an intraperitoneal injection of luteolin in APP23 mice. The ATF4- and CD68-positive microglia were significantly decreased in the brains of luteolin-injected mice when compared with DMSO-injected mice, suggesting the regulatory role of luteolin in ER stress and the suppression of microglial activation in the brain. Using the same APP23 mice model, the authors showed that the depressive behaviors of aged APP23 mice were improved by luteolin. Luteolin-stimulated memory impairment was confirmed by two experiments: the Y-maze spontaneous alternation test and the forced swimming test (FCT) [29].

In an AD mouse model obtained by the bilateral injection of amyloid-β_1–42_ oligomers into the CA1 region of the hippocampus, both an increase in the Aβ content and the activation of astrocytes and microglia in the hippocampus and cortex were observed. In the same AD model, the level of autophagy was decreased. The application of luteolin at a dose of 100 mg/kg/d reversed this state [30]. Luteolin also improved the brain histomorphology and reduced protein plaques (Aβ and p-Tau) in 3 × Tg-AD mice [27]. The reduction in Aβ deposits in the presence of luteolin was also observed in the APP23 model [29].

It was found that luteolin, in a concentration-dependent manner, attenuated the LPS-induced decrease in [(3)H]dopamine uptake and loss of tyrosine hydroxylase-immunoreactive neurons in primary mesencephalic neuron–glia cultures. The protective action of luteolin against the LPS-induced injury of dopaminergic neurons is associated with its efficiency in inhibiting microglial activation. This can explain the protective role that luteolin plays in Parkinson’s disease [31].

As luteolin is an anti-oxidant, its neuroprotective properties can result from inhibiting H_2_O_2_, NO, and malondialdehyde production and normalizing the activities of glutathione S-transferase and superoxide dismutase (SOD), which was confirmed in Wistar rats administered with cobalt chloride [32].

Some reports indicate that luteolin can suppress mast cell activation and reduce the release of various inflammatory mediators, e.g., histamine and tryptase, in neuroinflammatory conditions in vivo and in vitro [26].

Despite the poor oral bioavailability of luteolin (<2%) and its short half-life in plasma (<4 h) [33], the systematic intake of luteolin prevents or delays the onset, progression, and severity of AD and PD pathogeneses.

#### 3.1.3. Diosmetin (5,7,3′-Trihydroxy-4′-methoxyflavone)

Diosmetin, an O-methylated flavone, is found in the Caucasian vetch. Diosmetin is known as an anti-inflammatory, estrogenic, anti-oxidant, anti-microbial, and anti-cancer compound [34]. The results show that the modulation of the PI3K/AKT/NF-kB signaling pathway is one of the mechanisms by which diosmetin prevents neuroinflammation and neuronal apoptosis. Diosmetin treatment significantly reduces the levels of pro-inflammatory cytokines in the hippocampus in a rat model of bacterial meningitis (using the standard strain of *S. pneumoniae* serotype 3) when compared with the negative control group. It also reduces the levels of phosphoinositide 3-kinase (PI3K) and AKT protein (protein kinase B, PKB, serine/threonine-specific protein kinases) in the hippocampal tissue in the rat model [35].

Other studies have shown that treatment with diosmetin reduces the activity of the MyD88/NF-kB signaling pathway in the hippocampal tissue of the rat model of S. pneumonia-induced meningitis [36].

It is known that neurodegeneration, including the degeneration of dopaminergic cells, is strongly influenced by oxidative stress. Oxidative stress is related to other pathological conditions like mitochondrial dysfunction, neuroinflammation, and excitotoxicity induced by nitric oxide. The in vitro experiments showed that diosmetin interacted with the binding sites of several cellular target molecules associated with oxidative stress, e.g., adenosine A2A (AA2A) receptor, peroxisome proliferator-activated receptor gamma (PPARγ), protein kinase AKT1, nucleolar receptor NURR1, liver-X receptor beta (LXRβ), monoamine oxidase-B (MAO-B), and tropomyosin receptor kinase B (TrkB). Diosmetin possibly acts on several target receptors linked to the pathophysiology of PD, demonstrating promise as an oxidative stress inhibitor [37].

The monoamine oxidase (MAO) inhibitors have been used clinically to treat depression, anxiety, and Parkinson’s disease. Among the MAO inhibitors, there are reversible and selective MAO-A inhibitors, reversible and selective MAO-B inhibitors, and irreversible MAO-A and MAO-B inhibitors. Diosmetin was found to be a reversible and selective MAO-B inhibitor [38]. Both MAO-A and MAO-B are responsible for the oxidation of biologically active monoamines, including dopamine. Increased MAO-B activity is responsible for the decreased dopamine levels in the SNpc region of the mid-brain, which is remarkably associated with the pathogenesis of PD-like manifestations [39]. The inhibition of MAO-B by diosmetin can be potentially applied in PD treatment due to the enhanced striatal neuronal response to dopamine.

There are some reports that suggest a protective role for diosmetin in Alzheimer’s disease. Diosmetin reduced Aβ1–40 and Aβ1–42 production in CHO/APP695 cells and primary neuronal cells in a dose-dependent manner and also reduced γ-secretase activity in both cell types. It was found that diosmetin also enhanced the level of inhibitory phospho-GSK-3β (Ser9) and reduced tau phosphorylation in HeLa/tau cells. Densitometry analysis showed a significant increase in the ratio of pGSK-3β (Ser9) to total GSK-3β and a decrease in phosphorylated tau (p-tau) to total tau in diosmetin-treated HeLa/tau cells compared to the control samples without tested flavone. The same work showed that diosmetin dose-dependently reduced IFNγ-induced CD40 expression and the IFNγ/CD40L-induced production of pro-inflammatory cytokines (TNF-α and IL-12 (p70)) in primary microglial cells, which suggests that diosmetin reduces pro-inflammatory microglial activation. Additionally, diosmetin enhanced Aβ phagocytosis [40]. Similar results were obtained using the precursor of diosmetin, i.e., diosmin; thus, diosmin and diosmetin could be considered as potential candidates for anti-AD therapy [40].

Aβ peptide, considered to be one of the key proteins responsible for AD pathology, is formed from the amyloid precursor protein (APP). The APP undergoes sequential proteolytic cleavage by the coordinated actions of the membrane-embedded α-, β-, and γ-secretases with the generation of intermediates such as CTFα and CTFβ. Aβ peptides differ in length, and their most common isoforms are Aβ38, Aβ40, and Aβ42. Although Aβ40 is highly abundant (~90% of total Aβ), Aβ42 seems to be more toxic [41]. ApoE4 has been well documented to induce APP processing and Aβ production in in vitro experimental models of AD. It was demonstrated that diosmetin decreased the ApoE4-induced secretion of Aβ40 (extracellular Aβ40) in a dose-dependent manner when compared to ApoE4-treated rat neuroblastoma B103 cells that stably expressed human wild-type APP 695 isoforms (B103hAPP695wt). The production of intracellular Aβ40 in the ApoE4-treated B103-hAPP695wt cells was significantly increased when compared to that observed in vehicle-treated cells, whereas the pretreatment with diosmetin also significantly reduced the intracellular Aβ40 and Aβ42 levels when compared to those observed in ApoE4-treated cells. Additionally, diosmetin showed a potent ability to reduce Aβ levels without a significant increase in CTFs. These results suggest that diosmetin treatment prevented the ApoE4-induced APPs from being processed into Aβ peptides based on the α-secretase level [41].

The inhibitory effects of diosmetin on APP metabolism and Aβ generation were examined in another study performed on SH-SY5Y cells (a well-characterized cellular model of AD). Diabetes may be a risk factor for AD development. Hyperglycemia facilitates the glycosylation of proteins, with glucose residues resulting in the formation of advanced glycation end products (AGEs). Furthermore, AGEs activate a cascade comprising the production of reactive oxygen species and the stimulation of APP processing pathways, finally resulting in Aβ production. The aggregated Aβ results in ER stress, which initiates a multistep cascade, leading to neuronal death and dementia [42]. It was demonstrated that AGE-induced APP degradation and the increased production of Aβ were reversed by the presence of diosmetin in SH-SY5Y cells. The mechanism of action of diosmetin involved the inhibition of ER sensitivity to AGEs by the reduction in ROS generation. It was observed that diosmetin reversed the AGE-induced inhibition of anti-oxidative enzyme activity (SOD, CAT, GSH-Px), resulting in lowered ROS level, ER stress, and ER stress-mediated apoptosis. Diosmetin inhibited the next step of Aβ generation by decreasing the level of β-secretase-1, an enzyme that initiates APP processing. Finally, it was demonstrated that diosmetin enhanced the protein levels of two proteases, insulin-degrading enzyme (IDE) and NEP, involved in Aβ degradation. In this way, diosmetin protects against Aβ deposition [42].

#### 3.1.4. Apigenin (4′,5,7-Trihydroxyflavone)

Apigenin naturally occurs in many fruits and vegetables, but parsley, celery, celeriac, and chamomile tea are its best sources. It is known as an anti-oxidant, anti-inflammatory, anti-tumor, and neuroprotective compound.

The potential mechanism of action of apigenin was assessed in the transgenic Drosophila model of PD (transgenic fly lines that express wild-type human synuclein). The GSH content was significantly lower and the GSH activity was higher in PD flies compared to control flies. The PD flies exposed to apigenin showed a significant dose-dependent increase in the GSH (reduced glutathione) content and a significant dose-dependent decrease in the GSH activity, and the effect was similar to that observed after dopamine administration; however, the concentration of apigenin required to produce these effects was lower than the concentration of dopamine. Apigenin reversed the increased lipid peroxidation in PD flies, and PD flies exposed to apigenin showed a significant dose-dependent decrease in the MAO activity compared to unexposed PD flies. The activity of caspase-3 and caspase-9 was significantly lower in PD flies exposed to apigenin compared to unexposed PD flies. Finally, the authors suggested that apigenin can act as a MAO inhibitor, an anti-apoptotic agent, and a ROS scavenger, can restore the dopamine level, and thus mitigate PD symptoms.

The anti-inflammatory and neuroprotective properties of apigenin were studied using the co-culture model of neurons and glial cells obtained from the brain of Wistar rats. The glia/neurons were exposed to LPS or IL-1β to induce an inflammation process. To verify the neuroprotective potential of apigenin against neuroinflammation, the presence of active caspase-3, a classical apoptosis marker, and β-tubulin III was detected. It was observed that apigenin treatment significantly reduced the proportion of caspase-3-positive cells and caspase-3/β-tubulin III-positive neurons both in cells with LPS-induced and IL-1β-induced inflammation, when compared to cultures treated with LPS or IL-1β alone. Additionally, apigenin treatment caused a reduction in IL-6 in IL-1β-induced inflammation cells when compared to cells with IL-1β treatment alone. Moreover, apigenin was able to induce increased brain-derived neurotrophic factor (BDNF) mRNA levels in the neuronal and glial cell co-cultures after IL-1β-induced damage when compared to the IL-1β-treated group. It was found that the treatment with apigenin preserved neuron and astrocyte integrity, reduced microglial activation by the inhibition of proliferation, and decreased the expression of the M1 inflammatory marker CD68 in neurons and the glial cell co-culture model. The above data indicate that apigenin exerts neuroprotective and anti-inflammatory properties in vitro [43].

The protective role of apigenin in Parkinson’s disease was studied using a rat model of PD induced by rotenone, and the loss of tyrosine hydroxylase immunoreactivity in the striatum and substantia nigra was observed. Apigenin treatment significantly reversed the rotenone-induced upregulation of NF-κB gene expression and the rotenone-stimulated increase in pro-inflammatory mediators like pro-inflammatory cytokines, such as TNF-α and IL-6, and pro-inflammatory enzymes such as iNOS-1. Apigenin also prevented the reduction in neurotrophic factor mRNA expression, such as that of BDNF and glial cell line-derived neurotrophic factor (GDNF), as well as reducing α-synuclein aggregation and upregulating the protein expression of tyrosine hydroxylase and dopamine D2 receptor in rotenone-treated animals. All these findings indicate the protective role of apigenin in this animal model of Parkinson’s disease [44].

Similar results were obtained using the PD mouse model induced by MPTP. In this study, apigenin application reversed the MPTP-induced changes in protein expression and levels in the brain of neuroinflammatory agents like TNF-α, IL-1β, IL-6, IL-10, and TGF-β [45].

The other model of Parkinson’s disease is a cell model using MPP^+^-induced MES23.5 cell injury. MES23.5 dopaminergic cells display some similarities with primary neurons derived from the substantia nigra. MPP^+^, a monoaminergic neurotoxin, exerts toxic properties through several pathways involved in oxidative stress, apoptosis, and the inactivation of the PI3K/AKT cascade. The obtained results showed that apigenin inhibited the MPP^+^-induced translocation of the NF-κB p65 subunit and the MPP^+^-induced increase in the caspase-3 activity, and, in this way, it mediated apoptosis, inhibited NF-κB p65 phosphorylation, and inhibited the expression of inflammatory factors such as TNF-α, IL-1β, and IL-6 through the activation of the PI3K/AKT pathway. The addition of LY294002, an inhibitor of PI3K, may attenuate the suppressive activity of apigenin on inflammatory mediator levels [46,47].

Apigenin reduces the intracellular ROS content and, in turn, considerably increases the GSH concentration, thus improving the anti-oxidant defense system.

### 3.2. Flavonols

#### 3.2.1. Quercetin (3,3,4,5,7-Pentahydroxyflavone)

The other polyphenol with anti-inflammatory and anti-oxidative properties is quercetin, a flavonol commonly present as a glycoside, which is naturally found conjugated with residues of sugars. The quercetin family is divided into several classes depending on the absence or presence of additional groups, e.g., glycoside, sulfate, methyl-ether, or glucuronide forms [48,49]. Quercetin is widely distributed in nature and is found in many fruits (mainly berries), vegetables, leaves, seeds, and grains, but its bioavailability is rather poor. It is believed that the carbohydrate derivatives of quercetin, e.g., hyperoside (quercetin-3-O-β-D-galactoside) or aglucones, increase its bioavailability [48]. Quercetin is used as a dietary supplement in both free and conjugated states, and 1 g/day is believed to be its safe dose, considering an absorption of up to 60% [50]. Quercetin exerts its properties via several mechanisms. Cancer, allergic reactions, arthritis, and cardiovascular disorders are targets of quercetin applications. Quercetin is also involved in platelet aggregation and the peroxidation of lipids. Some studies confirm its role in the prevention of neurodegenerative diseases [49].

The neuroprotective properties of quercetin comprise both anti-oxidative and anti-inflammatory actions. It reduces the neuronal damage due to the stimulation of superoxide dismutase and catalase, the regulation of GSH level, and the downregulation of MDA level, as well as by being a scavenger of both ROS and RNS. An anti-inflammatory effect is exerted by increasing the IFNγ cell expression and decreasing the IL-4-positive cell expression [44]. Quercetin affects the expression of anti-oxidant enzymes, such as SOD and GSH-Px, in the brain [51], which is consistent with the quercetin-initiated downregulation of inflammatory cytokines and apoptotic markers.

In the MPTP-induced PD mouse model with the loss of DA neurons and activation of glia in the substantia nigra compacta, the conjugated quercetin derivative, hyperoside, inhibited the MPTP-induced activation of glia and downregulated the secretion of inflammatory factors, saving DA neurons. The mechanism of action of hyperoside involved the reduction in the expression of NLR Family Pyrin Domain-Containing 3 (NLRP3), apoptosis-associated speck-like protein containing caspases recruitment domain (ASC), and caspase-1 and an increase in the PACAP content in the substantia nigra of the mice. Pituitary adenylate cyclase-activated peptide (PACAP) is an endogenous neuropeptide with neuroprotective effects in various neurodegenerative diseases [52].

In the rotenone-induced PD mouse model, quercetin appears to be an anti-inflammatory agent that reversed the rotenone-caused increase in inflammatory markers in the serum, activated astrocytes in the substantia nigra and hippocampus, and decreased the density of dopaminergic fibers in the striatum. The protective role of quercetin was confirmed by behavioral tests like the Y-maze and T-maze tests [53].

Manganese (Mn) is an essential trace element that is required for the action of many enzymes in the body (including anti-oxidative enzymes) at physiological concentrations [54]. A pathological increase in Mn level can lead to the toxic accumulation of Mn in brain cells, resulting in the development of manganism. This is a neurodegenerative disorder similar to Parkinson’s disease in terms of damage and symptoms [55]. Mn can induce ROS generation, which significantly affects mitochondrial function, stimulates ER stress, and promotes apoptosis [56]. Dopaminergic SK-N-MC human neuroblastoma cells were used as an experimental model of Mn-induced neuroinflammation. It was observed that quercetin significantly decreased the intracellular ROS level in Mn-pretreated cells and increased the SOD and CAT activity and intracellular glutathione level. Pretreatment with Mn alone increased the levels of P-IκBα, NF-κB P65, HO-1, and Nrf2 proteins. This was not observed in the control group without Mn and quercetin. After quercetin administration, the levels of P-IκBα and NF-κB P65 markedly decreased, while a further increase in the levels of HO-1 and Nrf2 was noted. These results were confirmed in the same experiment using a Sprague Dawley male rat brain. Additionally, quercetin markedly decreased the protein expression of inflammatory markers such as TNF-α, IL-1β, IL-6, COX-2, and iNOS (induced by Mn administration) in the rat brain, and the expression of these markers was restored to the normal level. Furthermore, after Mn administration, the expression of the anti-apoptotic protein Bcl-2 significantly decreased while the expression of proteins like Bax, cytochrome c, cleaved caspase-3, and PARP-1 was higher. All these Mn-induced changes were reversed after quercetin treatment [57].

The murine BV2 microglial cells were used to study the anti-inflammatory activity of quercetin and its effect on the LPS-induced NO level and the Nrf2/HO-1 pathway. It was demonstrated that quercetin potently inhibited the LPS-induced NO level, and quercetin pretreatment resulted in a significant increase in the Nrf2 and HO-1 protein expression both in murine BV-2 microglial cells without LPS treatment and in cells after LPS exposure. The induction of phosphorylated p38MAPK and the increase in ERK1/2 activation induced by quercetin suggested the involvement of the MAPK/ERK1/2 pathway in quercetin’s action, especially as this effect was abolished in the presence of SB202190, a specific inhibitor of p38MAPK, and U0126, a specific inhibitor of mitogen-activated protein kinase kinase (MEK1/2), leading to the phosphorylation of ERK1/2 [58].

Studies indicate that quercetin attenuated amyloid-induced cytotoxicity and apoptosis when added to primary neuron cell cultures. Additionally, protein oxidation and lipid oxidation were reduced in the presence of quercetin [54]. The modulation of Nrf2 pathways might be an effective approach for reducing Aβ and tau hyperactivation; thus, it was confirmed that quercetin, through the modulation of Nrf2 as well as due to its anti-oxidative properties, plays an important role in protection against Alzheimer’s disease [59].

The increased accumulation of Aβ in the mouse brain is associated with a high-cholesterol diet that induces oxidative stress and inflammatory response. The oral administration of quercetin significantly improved the behavioral performance of high-cholesterol-fed old mice in both a step-through test and the Morris water maze task. The mechanism of action of quercetin comprises the increase in Cu/Zn-SOD activity and a subsequent decrease in the intracellular ROS level. Additionally, quercetin inhibited protein phosphatase 2C, which resulted in the activation of the AMP-activated protein kinase (AMPK), a reduction in iNOS and COX-2 expression, and the suppression of NFκB p65 nuclear translocation. This cascade lowered the expression of inflammatory markers such as IL-1β, IL-6, and TNF-α. Activated AMPK phosphorylated HMG-CoA reductase and acetyl-CoA carboxylase, and they lost their activity. Finally, low cholesterol levels, reduced Aβ-converting enzyme 1 (BACE1, α-secretase) expression, and lower Aβ deposits were observed [60].

Experiments performed in vitro on 6-hydroxydopamine (6-OHDA)-treated PC-12 cells or in vivo on a 6-OHDA-treated rat model of PD showed reduced oxidative stress, higher levels of the mitophagy markers PINK1 and Parkin, lower mitochondrial damage, and reduced expression and accumulation of the α-synuclein protein in the presence of quercetin. Additionally, in vivo results have shown that quercetin administration improves 6-OHDA-induced progressive PD-like motor behavior in PD rats [59].

All described results indicate the potent protective role of quercetin against both Alzheimer’s and Parkinson’s diseases.

#### 3.2.2. Myricetin (3,3′,4′,5,5′,7-Hexahydroxyflavone)

Myricetin, a flavanol found in vegetables, tea, fruits, berries, red wine, and medical plants, reportedly has anti-oxidant, anti-cancer, antiviral, anti-inflammation, antidiabetic, and antiatherosclerotic pharmacological actions [16]. Recently, the neuroprotective effects of myricetin were demonstrated. Myricetin was reported to attenuate neuronal injury by anti-oxidative and anti-inflammatory actions and to decrease LPS-induced inflammation by inhibiting the MAPK signaling pathway.

One of the animal models of AD are adult female Tg2576 mice that express a 695-aa residue splice form of human amyloid precursor protein modified by the Swedish familial AD double mutation K670N-M671L. The results showed that the oral administration of myricetin prevented the development of AD pathology by inhibiting the Aβ aggregation and oligomerization (the level of Aβ40 was significantly decreased) in this AD transgenic mouse model [61].

As demonstrated by another study, the inhibitory effect of myricetin on the accumulation of misfolded proteins inside or outside the neuronal cells involved modulating the endogenous levels of Hsp70 chaperone and quality control (QC)-E3 ubiquitin ligase E6-AP, an enzyme responsible for targeting proteins for degradation within proteosomes. This effect was abolished by the addition of a putative proteasome inhibitor (MG132) [62].

In the other study, the effect of myricetin was assessed in Aβ42 oligomer-treated neuronal SH-SY5Y cells and the 3 × Tg mouse AD model. The results of behavioral tests (e.g., the Morris water maze test) presented improvements in spatial cognition, learning, and memory in 3 × Tg mice after two weeks of an intraperitoneal administration of myricetin compared to the non-treated control group. It was found that myricetin prevented Aβ42O-induced p-TauS396, p-TauS356, and p-TauT231 hyperphosphorylation in SHSY5Y cells and 3 × Tg mice. Treatment with myricetin completely prevented the Aβ42O-induced reduction in presynaptic Synaptosomal-Associated Protein, 25kDa (SNAP25), expression and synaptophysin expression and also ameliorated the Aβ42O-induced reduction in postsynaptic density protein 95 (PSD95) expression in SH-SY5Y cells. These results were confirmed in the hippocampi of 3 × Tg mice. The mechanism of action of myricetin involved the decrease in the phosphorylation of ERK2 and GSK3β, in both in vitro and in vivo studies. Additionally, myricetin treatment completely reversed Aβ42O-induced ROS production, lipid peroxidation, and DNA oxidation (the determination of the levels of 8-OHdG and 4-HNE) in SH-SY5Y cells and the hippocampal brain tissue of 3 × Tg mice [63]. 8-hydroxy-2′-deoxyguanosine (8-OHdG) is a good biomarker for the endogenous oxidative damage of DNA and the risk assessment of various cancers and degenerative diseases [64], while 4-Hydroxynonenal (4-HNE) is one of the main products of lipid oxidation and PUFA peroxidation, and it is a highly reactive molecule involved in human disease development, including in the development of Alzheimer’s disease [65].

It was found that myricetin downregulated inflammatory processes. While microglial BV2 cells exposed to LPS showed significant NO generation and increased iNOS and COX-2 levels compared to control cells, pretreatment with myricetin inhibited this LPS effect. Myricetin also abolished the LPS-induced increase in TNF-α, IL-1β, and PGE_2_ levels, resulting in a reduction in pro-inflammatory modulators and cytokine levels. The mechanism of action of myricetin comprised the inhibition of the MAPK signaling pathway, which is normally activated during inflammation processes. Treatment with LPS significantly increased phosphorylated MAPKs, i.e., p-ERK, c-Jun NH2-terminal kinase (p-JNK), and p-38 levels, compared with the control cells, while myricetin decreased the LPS-induced components of mitogen-activated protein kinase signaling pathway levels [66].

Ionized calcium-binding adaptor molecule 1 (IBA-1) is a microglia/macrophage-specific calcium-binding protein whose levels increase in activated microglia. In in vivo experiments, the levels of IBA-1 were significantly increased in the hippocampi and cortices of the LPS-treated group of mice, which indicated high microglial activation. Myricetin administered intraperitoneally suppressed this increase in microglial activation in both areas of the mouse brain (the number of activated microglia was measured). These results suggest that myricetin regulates neuroinflammation by suppressing microglial activation [66].

Under some conditions, microglia rapidly transform from the resting state to the active pro-inflammatory M1 phenotype marked by an increased production of inflammatory cytokines (e.g., IL-6, TNF-a, and IL-1β) and reactive oxygen and nitrogen species (ROS/RNS), leading to the increase in oxidative stress and neuronal degeneration [67]. Microglial activation involves various signal transduction pathways, including Nrf2, activator protein-1 (AP-1), NF-κB, and signal transducer and activator of transcription 1 (STAT1). In an in vitro model using the neuroblastoma cell line SHSY5Y and hypoxia-activated murine microglia BV-2 cells, it was found that myricetin can switch off M1 microglia polarization through the inhibition of STAT1 signaling by the direct interaction of myricetin with STAT1, resulting in the reduction in its S-glutathionylation and Tyr701 phosphorylation [68].

Myricetin mitigated MPTP-induced motor impairment in the PD rat model using the foot-fault test. To assess the roles of myricetin in regulating dopamine neuronal loss and α-synuclein accumulation in the SN of PD models, the expression of tyrosine hydroxylase (TH) and α-synuclein was measured. Results showed that myricetin inhibited the MPTP-induced increase in α-synuclein protein levels. TH protein expression was restored by myricetin, and TH-positive cells were increased in the SN after myricetin administration in MPTP-treated rats. All these effects were blocked by erastin, a ferroptosis activator [69].

The iron overload is a pathological hallmark of PD. Ferroptosis is an iron-dependent cell death form characterized by ROS overgeneration and lipid peroxidation. The Fe^2+^ levels in the SN and serum were markedly increased after MPTP treatment, whereas myricetin effectively inhibited this increase. Myricetin also reversed the MPTP-induced ROS level and the MPTP-caused decreases in GSH levels in the SN. The roles of myricetin in regulating ferroptosis were verified in the in vitro MPP^+^-SH-SY5Y cell model of PD. The obtained results demonstrated that myricetin alleviates MPP^+^-induced SH-SY5Y cell ferroptosis (Fe^2+^, ROS, and GSH levels). Additionally, myricetin accelerated the nuclear translocation of Nrf2 and the subsequent glutathione peroxidase 4 (GSH-Px4) expression in MPP^+^-treated SH-SY5Y cells, the two critical inhibitors of ferroptosis. Data demonstrate that myricetin may be a potential protective agent against dopaminergic neuron death by inhibiting ferroptosis in PD [69].

Myricetin was reported to have an inhibitory action on the excessive glutamate release, which leads to neurological disorders by decreasing K^+^ channel blocker 4-aminopyridine and voltage-dependent Ca^2+^ entry in rat cerebrocortical nerve terminals [70].

### 3.3. Flavanols

#### Catechins (Flavan-3,3′,4′,5,7-pentol) (2R,3S)-2-(3,4-Dihydroxyphenyl)-3,4-dihydro-2H-chromene-3,5,7-triol)

The group of catechins (flavan-3-ol) includes (−)-epigallocatechin-3-gallate (EGCG), (−)-epicatechin-3-gallate (ECG), (−)-epigallocatechin (EGC), and (−)-epicatechin (EC). The best source of catechins is unfermented green tea, but catechins also occur naturally in black tea, coffee, cinnamon, berries, grapes, and wine. Anti-inflammatory, anti-oxidant, and chemopreventive activities are considered the most important actions of catechins [71].

As mentioned previously, obesity and a high-fat diet can be causes of neuroinflammation, which leads to microglial activation. The effects of (−)-epigallocatechin gallate (EGCG) on the generation of pro-inflammatory cytokines were studied using palmitic acid (PA)-stimulated BV-2 microglia and high-fat-diet-induced obese male C57BL/6J mice. The accumulation of lipid droplets in PA-treated BV-2 cells was increased compared to that in the control but was clearly improved in groups with EGCG pretreatment. In addition, results showed lower levels of TNF-α, IL-6, and IL-1β in the EGCG-treated cells. The mechanism of action of EGCG comprised the prevention of phosphorylation of JAK2 and STAT3, which is markedly increased in PA-treated cells compared to the control group. This suggests that JAK2/STAT3 signaling may be an important and effective target for EGCG in PA-stimulated BV-2 cells. EGCG-treated obese mice significantly restored the blood levels of glucose, total cholesterol (TC), and triglycerides (TGs) under fasting conditions. Moreover, in the EGCG-treated group, a decline in the inflammatory cytokine levels (TNF-α, IL-6, and IL-1) was found. The EGCG-untreated high-fat-diet group showed a higher microglial activation marker (Iba-1) level in the hypothalamus than that in the EGCG-treated group. An in vivo study confirmed the role of the JAK2/STAT3 signaling pathway in the effect of EGCG [72].

A similar effect of EGCG was observed on the inflammatory marker level in microglia BV2 cells, where inflammatory microenvironments were induced by simultaneous exposure to lipopolysaccharide (LPS) and the amyloid-β oligomer (AβO). EGCG reduced the LPS/AβO-induced inflammation, which was confirmed by the measurement of IL-1β, IL-6, and TNF-α levels. EGCG reduced the activation of the NOD-, LRR-, and pyrin domain-containing protein 3 (NLRP3) inflammasome as well as the levels of intracellular ROS in BV2 cells treated with LPS/AβO by affecting the mitochondrial membrane potential (MMP) (a lower level of thioredoxin-interacting protein (TXNIP) was demonstrated) [73].

An improvement in cognitive impairment (using the Morris water maze test) after ECGC administration was also observed in the rat model of AD obtained by the injection of Aβ25–35 to the CA1 area of the bilateral hippocampus. The mechanism of action of catechins involved several pathways. ECGC reduced oxidative stress by the stimulation of SOD and GSH-Px activity, diminished BACE1 expression and activity and Aβ1–42 level, inhibited tau phosphorylation (at Ser396), and repaired cholinergic deficit by the inhibition of acetylcholine esterase and the increased concentration of acetylcholine [74].

EGCG pretreatment reduced anxiety-like behavior and motor impairments, validated by behavioral tests in the PD model of C57BL/6N mice with α-synuclein preformed fibril (α-syn-PFF)-induced neural damage. The increased pro-inflammatory cytokine (Il-1, IL-6, TNF-α) expression observed in the α-syn-PFF group was significantly reduced by EGCG treatment in the striatal and SN regions. Furthermore, EGCG treatment significantly increased the expression of the anti-inflammatory cytokines transforming growth factor-β (TGF-β), IL-10, and IL-4 in the SN and anti-inflammatory factors IL-10 and IL-4 in the striatum. In addition, the PFF-induced degeneration of TH-immunopositive neurons was reversed by EGCG treatment, and the suppressed accumulation of p-α-synuclein in the SN and striatum was observed [75].

In vitro studies showed that both EGCG and epicatechin decreased the caspase-3 level in SK-N-AS cells exposed to 6-OH-DO. Additionally, epicatechin reversed the 6-OH-DO-induced increase in IL-1β. No changes in TNF-α were observed. The obtained results suggested that epicatechin may be more effective in preventing damage to neurons in PD [76].

EGCG is known as a potent anti-oxidant, and it well documented that it enhances the activity of phase II enzymes and detoxification enzymes, including catalase, glutathione peroxidase (GSH-PX), superoxide dismutase (SOD), and glutathione S-transferase [77]. The reduction in oxidative stress leads to the inhibition of neuroinflammatory pathways. There are many reports that link the anti-oxidative and anti-inflammatory actions of catechins with neuroprotection against AD and PD via the modulation of the NF-κB/STAT1 pathway, the Nrf2 pathway, and pro- and anti-apoptotic factors [78].

### 3.4. Flavanone

#### Eriodictyol ((2S)-3′,4′,5,7-Tetrahydroxyflavan-4-one)

Eriodictyol is found in various medicinal plants, citrus fruits, and some vegetables. This flavanone is consumed through diet due to its anti-oxidant, anti-inflammatory, anti-diabetic, anti-obesity, anti-cancer, neuroprotective, cardioprotective, and hepatoprotective properties [79].

In relation to the anti-oxidative properties of eriodictyol, its effect on LPS-induced neuroinflammation was studied in vivo (LPS i.p. injected into C57BL/6J mice) and in vitro (LPS-treated microglial BV-2 cells). In the BV-2 cells, eriodictyol evidently prevented the LPS-stimulated overproduction of ROS and NO and prevented the inhibitory effects of LPS on the levels of GSH and the levels and activities of CAT and SOD. Additionally, the increase in the mRNA levels of manganese superoxide dismutase (MnSOD) and a small increase in γ-glutamylcysteine synthetase (γGCS) and GSH peroxidase levels were observed. Then, it was found that eriodictyol affected the components of the NF-κB and MAPK pathways, which are regulated by ROS in the LPS-triggered BV-2 cells. Eriodictyol further suppressed the expressions of COX-2, p-JNK, and p-p38. Eriodictyol also increased the levels of Nrf2 and the expression of HO-1 and NQO-1 in vivo and in vitro. Second messengers such as cAMP and Ca^2+^ are necessary to regulate the cell signal transduction and enzymatic activity in the intracellular metabolic system. It was found that the levels of cAMP and Ca^2+^ were clearly elevated in the LPS-induced cells, whereas preincubation with eriodictyol significantly inhibited this trend in BV-2 cells [80]. The results of another study using the same model of neuroinflammation showed that eriodictyol reduced the aggregation of Aβ1−42 when compared to the LPS-treated C57BL/6J mice by the inhibition of the expression of APP and BACE1; thus, it intervened in the generation of Aβ1−42. The activation of glial cells contributes to the release of pro-inflammatory factors. The mRNA and protein levels of TNF-α, Il-6, and IL-1β were markedly higher in the LPS-treated group than those in the control group, whereas the supplementation of eriodictyol remarkably inhibited them. The mechanism of suppression of inflammatory cytokine release by the tested flavonoid involved the reduction in the phosphorylation of IκB and NF-κB and the levels of COX-2, iNOS, and TLR4. MAPKs were one of the most important signals mediating the inflammatory response, and results showed that eriodictyol evidently decreased the expressions of p-P38, p-JNK, and p-ERK in the mouse brain [81]. Eriodictyol also equilibrated the cholinergic system via the reduction in the AChE activity and the increase in the ChAT activity, as well as the final acetylcholine level. Eriodictyol suppressed the LPS-induced glial overactivation and regulated inflammatory mediators and cytokines by inhibiting the NF-κB and MAPK pathways [81].

The inhibitory action of eriodictyol on Aβ accumulation and the participation of Nrf2 and JNK/p38 apoptotic signaling pathways in this process were also observed in the Aβ25–35-induced oxidative cell death in the primary neuron AD model [82].

Aβ plaques can stimulate inflammasome-related protein 3 (NLRP3) inflammasome, resulting in the generation of caspase-1-mediated interleukins, IL-1 and IL-18, in microglia, which further aggravates the development and progression of AD. It was found that eriodictyol and homoeriodictyol can improve Aβ25–35-induced memory impairment (confirmed by the Y-maze test and the NOR experiment) in mice (after an intracerebral injection of Aβ25–35) by modulating the NLRP3 inflammasome. Both flavonoids significantly decreased the levels of Aβ1–40, Aβ1–42, and p-Tau in the mouse hippocampus. Furthermore, the decreased level of ROS but the increased levels of SOD and GSH-Px were observed in mouse hippocampal cells treated with flavonoids when compared to those not treated with flavonoids. The protein expression levels of NLRP3, caspase-1, ASC, and the inflammatory factors IL-18 and IL-1 were also measured. It was found that eriodictyol and homoeriodictyol decreased both the tested protein expression levels and inflammatory factor concentrations [83].

Eriodictyol reduced Aβ aggregation and Tau phosphorylation and thus alleviated the cognitive dysfunction in the AD model with APP/PS1 mice. Decreased Tau hyperphosphorylation and neurotoxicity were also observed in HT-22 hippocampal cells induced by Aβ1–42 oligomer exposed to eriodictyol. The characteristics of ferroptosis iron aggregation, such as lipid peroxidation and the expression of glutathione peroxidase type 4 (GSH-Px4), were markedly lowered by treatment with eriodictyol both in vivo and in vitro. The results indicated that the mechanism was associated with the activation of the Nrf2/HO-1 signaling pathway [84].

### 3.5. Isoflavone

#### Biochanin (5,7-Dihydroxy-40-methoxyisoflavone)

Biochanin is an isoflavone that belongs to the phytoestrogen family. Due to its molecular structure and size being similar to those of estradiol, phytoestrogens, including biochanin A, exert pro-estrogenic or anti-estrogenic effects by binding to α and β estradiol receptors. Isoflavones occur primarily in legumes, soybeans, red and white clovers, and alfalfa. Together with other isoflavones, genistein, and daidzein, biochanin A is a component of dietary supplements and is also used instead of hormonal replacement therapy to mitigate menopausal symptoms as a safer choice [85]. Biochanin A possesses anti-inflammatory, anti-cancer, neuroprotective, anti-oxidant, anti-microbial, and hepatoprotective properties. It displays these properties by inducing apoptosis, inhibiting metastasis, arresting the cell cycle, and blocking the expression and activity of pro-inflammatory cytokines via the modulation of NF-κB and MAPKs. Biochanin A can also lower microglial activation or the apoptosis of neurons, thus showing neuroprotective features [86].

The inhibitory effects of biochanin A on LPS-induced pro-inflammatory responses by the inhibition of mitogen-activated protein kinase (MAPK) signaling pathways were observed in BV2 microglial cells. The LPS-induced increase in the mRNA and protein levels of pro-inflammatory cytokines TNF-α and IL-β1 was significantly decreased in the presence of biochanin A. Biochanin A reduced the mRNA level and protein expression of iNOS and the level of NO in BV-2-activated cells afterwards. LPS alone treatment stimulates the phosphorylation of JNK, ERK, and p38, but pretreatment with biochanin A prevents the LPS-induced pro-inflammatory cytokine production in BV2 microglial cells [87]. Biochanin A mitigated the LPS-induced decrease in dopamine uptake, the number of dopaminergic neurons, and microglial activation in rat mesencephalic neuron–glia cultures with the simultaneous inhibition of TNF-α, NO, and superoxide [88]. In vivo, biochanin A prevented the activation of microglia and dopaminergic neuron damage, as well as improved the behavioral symptoms of LPS-treated rats in the animal model. The inhibition of the phosphorylation of ERK, JNK, and p38 in the substantia nigra of PD rats and an increase in the SOD and GSH-Px activities in the rat brain were observed. Additionally, the levels of IL-1β, IL-6, and TNF-α in the serum were lower after biochanin A administration [89,90].

The less common mechanism of action of biochanin A involves the brain renin–angiotensin system, which is known as a modulator of the neuroinflammatory responses and the progression of dopaminergic degeneration. Angiotensin II (Ang II) induces microglial activation via angiotensin II type 1 receptor (AT1R), consequently affecting the function of dopaminergic neurons. Biochanin A treatment attenuated the behavioral dysfunction, inhibited the microglial activation, and prevented DA neuron damage in the Ang II-induced rat PD model. The reduction in NLRP3, caspase-1, and pro-inflammatory cytokines was demonstrated. The mechanism of action of biochanin A may involve an increase in the endophilin A2 (EPA2) expression and a decrease in the AT1R expression. As endophilin A2 is involved in the endocytosis of several G-protein-coupled receptors, the total amount of AT1R decreased in AngII-induced rats [91]. Since estrogen deficiency leads to mitochondrial defects that provoke Alzheimer’s disease-associated pathological changes in a postmenopausal mouse model, the neuroprotective abilities of biochanin A were studied in an AD in vivo model with ovariectomized (OVX) APP/PS1 mice. It was observed that Aβ deposition and BACE1 expression in the hippocampi of ovariectomized APP/PS1 mice are decreased in the presence of biochanin A, which can be a reason for the cognitive function improvement in these mice (confirmed by the behavioral test, e.g., the Morris water maze test). Flavonoids reversed the imbalance of mitochondrial dynamics and abnormal mitophagy caused by ovariectomy [92]. A similar effect of biochanin A was observed for learning and memory using the postmenopausal-like model of ovariectomized rats (also checked by the Morris water maze test); additionally, the activation of the intracellular anti-oxidant enzymes SOD and GSH-Px and the simultaneous inhibition of oxidative damage were confirmed. Results suggest that biochanin A can be helpful in protecting postmenopausal women against Alzheimer’s disease development [93].

Similarly to other described flavonoids, biochanin A can prevent neurodegeneration via the inhibition of ferroptosis. Biochanin A reduced the glutamate- or kainic acid-induced ferroptosis of mouse hippocampal neurons. The results showed that biochanin A significantly inhibited the accumulation of intracellular iron and lipid peroxidation but increased the level of GSH-Px4. Intracellular Fe^2+^ was decreased by regulating the expression of iron metabolism-related proteins: ferroportin-1, divalent metal transferase 1, and transferrin receptor 1 [94].

Biochanin A is potentially an anti-apoptotic agent and may be able to exert neuroprotective effects against glutamate-induced cytotoxicity in PC12 cells. The pretreatment of glutamate-treated PC12 cells led to decreases in the number of apoptotic cells and the activity of caspase-3 but an increase in the total glutathione level [95]. Pretreatment with biochanin A attenuated the cytotoxic effect of the Aβ25–35 protein when PC12 cells were exposed to Aβ25–35. These effects were achieved by decreasing the apoptotic rate (a significantly lower number of apoptotic cells) with the restoration of Bcl-2/Bax and Bcl-xL/Bax ratios in the presence of biochanin A. Biochanin A also suppressed the Aβ25–35-induced caspase-3, caspase-8, and caspase-9 activity. Since the expression of cytochrome c and p53-upregulated modulator of apoptosis (Puma) was reduced in biochanin A-treated cells, the results suggested the participation of mitochondria in the protective effect of biochanin A [96].

In docking studies, it was demonstrated that biochanin A is a potent, reversible, and selective human MAO-B inhibitor and forms H-bonds and hydrophobic interactions at the active sites of the enzyme. Biochanin A can also block the active site of MAO-A but with a significantly higher inhibitory constant (k_i_ for hMAO-B = 3.8 nM; k_i_ for hMAO-A = 99.3 nM) [97]. The inhibitory property of biochanin A on the BACE1 activity was evaluated using a fluorescence resonance energy transfer (FRET) assay (Pan Vera) with a recombinant baculovirus-expressed BACE1 and a specific substrate (Rh-EVNLDAEFK-Quencher). It was found that biochanin A acts as a BACE1 non-competitive inhibitor and binds equally well to the enzyme whether or not it is already bound to the substrate. Docking analysis confirmed that biochanin A might bind to BACE1 allosteric sites through hydrogen bond interactions. Thus, biochanin A can directly decrease the generation of Aβ, its aggregation, and Alzheimer’s disease development [98].

## 4. Discussion

**Figure 1:**
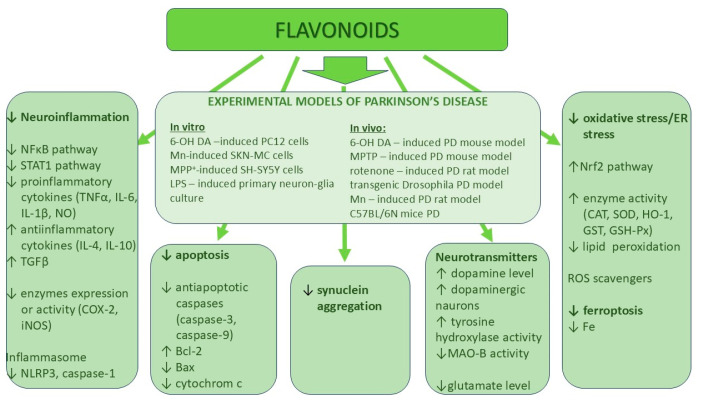
The potential mechanisms of neuroprotective actions of flavonoids in Parkinson’s disease (PD), based on the in vitro and in vivo models of PD. ↑—stimulation/increase; ↓—inhibition/decrease.

**Figure 2:**
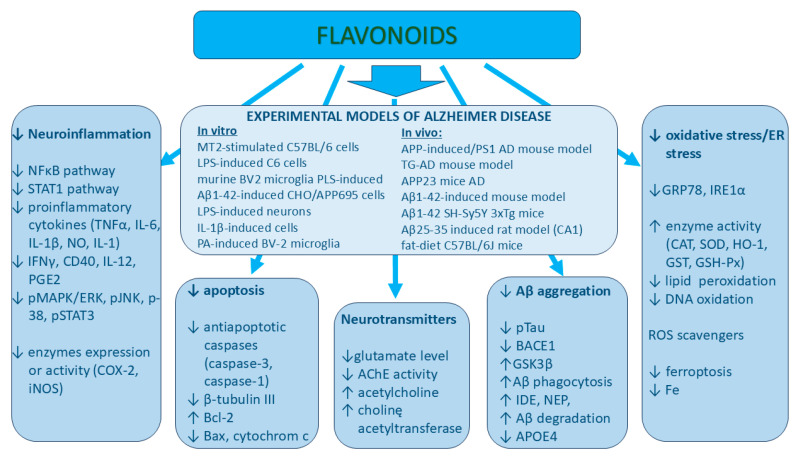
The potential mechanisms of neuroprotective actions of flavonoids in Alzheimer’s disease (AD), based on the in vitro and in vivo models of AD. ↑—stimulation/increase; ↓—inhibition/decrease.

This review is focused on the mechanisms of action of selected flavonoids by which these natural compounds can prevent the development of Alzheimer’s disease and Parkinson’s disease. The work presented in this review has some limitations. Flavonoids comprise over 9000 known compounds, and in this review, only the most common compounds are presented. The described flavonoids represent five subclasses of polyphenols, i.e., flavones, flavonols, flavanones, flavanols, and isoflavonoids, and are found in most popular dietary products (e.g., vegetables, fruits, and tea), which are present in people’s daily diet (examples of dietary products are listed in Table 1). The neuroprotective mechanisms of action of flavonoids in Parkinson’s disease and Alzheimer’s disease are presented in Figure 1 and Figure 2, respectively. Interestingly, all the above-mentioned flavonoids exert similar mechanisms of action; for example, all of them can inhibit neuroinflammation directly by affecting the NF-κB pathway by decreasing pro-inflammatory cytokines. Their other common mechanism of action comprises the activation of the Nrf2 pathway and the stimulation of anti-oxidative enzymes like CAT, SOD, GST, and HO-1. However, this mechanism was not observed after apigenin treatment. Some flavonoids such as luteolin, apigenin, and myricetin increase the tyrosine hydroxylase activity and the dopamine level in the brain, which is very important in the case of Parkinson’s disease, while a decrease in the BACE-1 activity by chrysin, catechins, quercetin, eriodyctiol, and biochanin A is crucial for reducing amyloid-β aggregation during the development of Alzheimer’s disease. Notably, each described flavonoid can exert more than one biological mechanism of action.

Experiments described in this review were performed with the use of animal (rat or mouse) or cell line models for both neurodegenerative diseases, and this is enough to recognize the cellular mechanisms of flavonoid action, but it cannot be a basis to plan therapeutic strategies in humans. The application of flavonoids as drugs for AD and PD treatment requires prolonged future studies. The number of clinical studies on humans is very limited due to several reasons. The list of problems comprises poor and not clearly known bioavailability, low knowledge about toxicity and side effects caused by high doses of pure flavonoids, the lack of safe dose recommendations, long time of observation, etc.

The bioavailability of flavonoids from one’s diet depends on different factors. First, the content of these phytochemicals in dietary sources can differ depending on the kind or variety of plants, environmental factors (e.g., soil types, sun exposure, dampness), climate, and types of culture. The other factors that influence the content of flavonoids in plants are the types of harvesting, storage conditions (temperature, humidity, and light accessibility), and the methods of culinary preparation—peeling (some flavonoids are found in the skin or close to the skin), cooking, and a high level of processing [99,100,101]. The content of flavonoids differs according to the subclass of polyphenol; for example, flavonols are found in a low concentration, i.e., 15–30 mg/kg wt, in food; the oil of mandarins is rich in flavones with a concentration of 6.5 g/L; and orange juice contains 200–600 mg/L of hesperidin [99]. The third important factor that affects bioavailability is the native form of flavonoids in food products. Polyphenols are better absorbed in the intestine than carotenoids, but their bioavailability is still poor. Most polyphenols, including flavonoids, are present in food in a form that cannot be absorbed: glycosides, esters, or polymers. They must be hydrolyzed in the intestine by intestinal enzymes or by colonic microflora before absorption [99]. Only aglycones and some glucosides can be absorbed in the small intestine in their unchanged form. All flavonoids except flavanols exist in their glycosylated forms, but the type of attached sugar (glucose, rhamnose, galactose, arabinose, or xylose) matters during absorption. For instance, in the case of quercetin, quercetin-4-glucoside is absorbed more efficiently than quercetin-3-rutinoside and quercetin aglycone [99,100]. Sometimes, other food components can reduce flavonoid bioavailability, e.g., milk proteins or dietary fibers; however, the consumption of flavonoids in the presence of lipids facilitates their absorption. After absorption, flavonoids are transported (carrier proteins may be required) to various tissues (including the brain) and then undergo biotransformation (methylation, sulfation, or glucuronidation) and secretion. The rates of these processes also determine the actions of flavonoids [99,100].

Modern techniques have significantly improved the absorption of flavonoids even after their oral administration. They improve the therapeutic efficacy, release control, and bioavailability of drugs by preparing drugs or carriers at the nanoscale. The combination of modern formulation techniques with flavonoids represents a promising area of research for improving their bioavailability. The new forms of drugs comprise nanosuspensions (e.g., luteolin), liposomes (e.g., quercetin), nanoemulsions (e.g., luteolin), etc. [13,101].

As mentioned above, colonic microbiota can increase flavonoid absorption. The relationship between gut microbiota and flavonoid bioavailability is bilateral. On the one hand, intestinal microbiota hydrolyzes the glycosidic forms of flavonoids to their aglycone forms, which can be absorbed; on the other hand, flavonoids modulate the structure of the gut microbiota population and reduce the growth of LPS-producing bacteria. Thus, flavonoids can act as prebiotics and can significantly protect the gut from inflammation by improving the integrity of the epithelial barrier and decreasing the production of pro-inflammatory cytokines in the colon and brain [99,102]. For instance, catechins modify the composition of gut microbiota by reducing pathogenic species such as *Helicobacter pylori*, *Escherichia coli*, or *Listeria monocytogenes* and by stimulating the growth of strains such as *Actinobacteria* and *Verrucomicrobia* [102].

Additionally, it was evidenced that the microbiota–gut–brain axis plays an important role in preventing the development of Parkinson’s disease and in improving the lives of PD patients. The primary production of serotonin takes place in gut cells in the presence of gut microbiota, which is improved by dietary flavonoids. Furthermore, the brain stem is connected to the enteric nervous system through the vagus nerve, which facilitates the brain–gut microbiota communication. The microbiota influences host behaviors like anxiety, eating, and depression through vagal neurons and the changes in brain neurotransmitters such as GABA and oxytocin. Moreover, in PD patients, the gut microbiota protects against gut inflammation and the accumulation of α-synuclein [102].

## 5. Conclusions

The aforementioned biological actions of flavonoids show that they can prevent the development of neurodegenerative diseases, such as Alzheimer’s disease and Parkinson’s disease, and can be considered as novel supportive agents in neurodegeneration treatment in the future. These plant-derived compounds offer a natural and potentially safe alternative to conventional drugs. Dietary flavonoids are rather safe (except at high doses, e.g., quercetin) and do not produce side effects. All the described mechanisms of action were studied in the presence of relatively low concentrations of flavonoids (5–100 μM). Dietary flavonoids exhibit poor oral bioavailability; however, a very wide array of flavonoids are present in various food products, and their similar mechanisms of action guarantee a sufficient supply of flavonoids to an organism for preventing diseases. Flavonoids can cross the blood–brain barrier and exhibit neuroprotective properties, affecting neurodegenerative progression. These properties of flavonoids were confirmed in both in vivo and in vitro studies using various AD and PD models. Evidently, members of all subclasses of flavonoids possess anti-oxidative, anti-inflammatory, and neuroprotective properties.

## Figures and Tables

**Table 1:** The characteristics of the flavonoids from different subclasses, described in brief in this review.

Class of Flavonoids	Name of Compound	Sources	Structure	Range of Effective Concentration/Dose	Affected Components Involved in Neuroprotection
Flavones	Chrysin	Honey, propolis, passion flowers, carrots, and chamomile	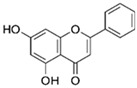	In vitro: 5–10 μM In vivo: Orally: 25 and 50 mg/kg for 4 days i.p. 25 mg/kg/day	↓NF-κB, ↓NO, ↓IL-6, ↓TNF-α, TRL-4, ↓COX-2, ↓iNOS, ↓MPO ↑IL-4, ↑IL-10 ↑Nrf2, ↑SOD, ↑CAT, HO-1 ↓Aβ, ↓p-Tau, ↓BACE-1, ↓GSK3β, ↓glutamate, ↓AChE
	Luteolin	Celery, broccoli, artichoke, green pepper, parsley, thyme, dandelion, perilla, chamomile tea, carrots, olive oil, peppermint, rosemary, navel oranges, and oregano	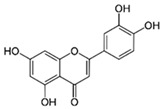	In vitro: 1–25 μM In vivo: i.p. 20, 100 mg/kg/d i.p. 20 mg/kg/d Orally: 30–100 mg/kg/day	↓NF-κB, ↓NO, ↓IL-6, ↓IL-β1, ↓TNF-α, ↓COX-2, ↓iNOS, ↓GRP78, ↓IRE1, ↓GFAP ↑Nrf2, ↑SOD, ↑GST ↓Aβ, ↓p-Tau, ↑dopamine, ↑tyrosine hydroxylase
	Diosmetin	Caucasian vetch	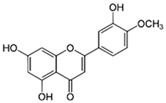	In vitro: 2.5–50 μM Orally: 100–200 mg/kg/day	↓PI3K/AKT pathway ↓NF-κB, ↓IL-6, ↓TNF-α, ↓IL-12 ↑Nrf2, ↑SOD, ↑CAT, ↑GSH-Px ↓Aβ, ↓p-Tau, ↑GSF-3β ↑Aβ phagocytosis, ↓ApoE4, ↓IFNγ, ↓CD40 ↓MAO-B, Trkβ ↑IDE, ↑NEP, ↑Aβ degradation
	Apigenin	Parsley, celery, celeriac, and chamomile tea	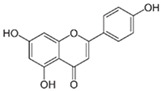	In vitro: 10–80 μM, In vivo: i.p. 10–50 mg/kg/day, Orally: 20 mg/kg/day for 30 days	PI3K/AKT ↓NF-κB, ↓IL-6, ↓TNF-α, ↓IL-1β, ↓iNOS, ↑BDNF, ↑GDNF ↑GSH, ↓lipid oxidation ROS scavenger ↑tyrosine hydroxylase ↑dopamine, ↓MAO-B ↓α-synuclein aggregation ↑caspase-3, ↑caspase-9
Flavonols	Quercetin	Capers, radish, red onion, radicchio, lovage, dock, and honey	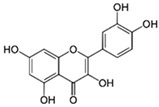	In vitro: 10–30 μg/mL In vivo: i.p. 10–100 mg/kg/day Orally: 30–50 mg/kg/day 60 days	MAPK/ERK1/2 ↓NF-κB, ↓IL-6, ↓TNF-α, ↓IL-1β, ↓COX-2, ↓iNOS, ↑Nrf2/HO-1, ↑SOD, ↑CAT, ↑GSH-Px, ↑GSH ROS scavenger ↓Aβ, ↓p-Tau, ↓BACE-1, ↓NLRP3, ↓caspase-3, ↓caspase-1, ↓Bax, ↑Bcl-2, ↓cytochrome c
	Myricetin	Vegetables, tea, nuts fruits, berries, red wine, and medical plants	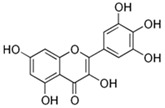	In vitro: 10–50 μM In vivo: i.p. 10–100 mg/kg/day i.g. 25 mg/kg/day	↓pMAPK/ERK1/2, ↓pJNK, ↓pp38, ↓IBA-1 ↓NF-κB/STAT1 ↓IL-6, ↓TNF-α, ↓IL-1β, ↓COX-2, ↓iNOS, ↓PGE2 ↑Nrf2, ↑GSH-Px, ↑GSH ↓Fe^2+^, ↓ferroptosis ↓Aβ, ↓GSK-3β, ↓α-synuclein, ↑tyrosine hydrolase, ↓glutamate ↓DNA oxidation ↓lipid oxidation
Flavanol	Catechins	Tea, cacao, pome fruits, and wine	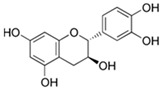	In vitro: 5–20 μM	↓NF-κB/STAT1, ↓pSTAT3, ↓pJak2 ↓IL-6, ↓TNF-α, ↓IL-1β, ↑Nrf2, ↑SOD, ↑CAT, ↑GSH-Px, ↑GSH ↓Aβ, ↓p-Tau, ↓BACE-1, ↓AChE, ↓caspase-3, ↑IL-10, ↑IL-4, ↑TGF-β
Flavanone	Eriodictyol	Yerba santa, citrus fruits, and peanuts	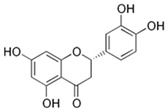	In vitro: 50 μM In vivo: i.g. 25–100 mg/kg/day	↓NF-κB/MAPKs, ↓pERK, ↓pJak2, ↓p-p38, ↓IL-6, ↓TNF-α, ↓IL-1β, ↓COX-2, ↓iNOS ↑Nrf2/HO-1, ↑SOD, ↑CAT, ↑GSH-Px, ↑GSH, ↑GCS ↓ferroptosis ↓Aβ, ↓p-Tau, ↓BACE-1, ↓APP, ↓TRL4 ↓AChE, ↑ChAT ↓caspase-1, ↓IL-1, ↓IL-18
Isoflavone	Biochanin A	Soya, red clover, alfalfa, chickpeas, fruits, vegetables, and nuts	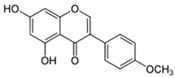	In vitro: 1.25–5 μM	↓NF-κB/MAPKs, ↓pERK, ↓pJNK, ↓p-p38, ↓IL-6, ↓TNF-α, ↓IL-1β, ↓COX-2, ↓iNOS ↑Nrf2, ↑SOD, ↑CAT, ↑GSH-Px, ↑GSH, ↓Fe^2+^, ↓ferroptosis ↓Aβ, ↓BACE-1, ↓MAO-B ↓NLRP3, ↓caspase-1, ↓caspase-3, caspase-8, caspase-9, ↓Bax, ↑Bcl-2 ↓cytochrome ↑EPA2 and ↓AT1R

↑—stimulation/increase; ↓—inhibition/decrease.

## Data Availability

Not applicable.

## Abbreviations

The following abbreviations are used in this manuscript:

CNS	central nervous system
PD	Parkinson’s disease
AD	Alzheimer’s disease
Aβ	amyloid-β
NF-κB	nuclear factor kappa-light-chain-enhancer of activated B cells
ROS	reactive oxygen species
TNF-α	tumor necrosis factor α
NO	nitrate oxide
NOS	nitrate oxide synthase
IL-	interleukin-
LPS	lipopolysaccharide
TRAF	TNF receptor-associated factor
6-OHDA	6-hydroxydopamine
COX-2	cyclooxygenase-2
CAT	catalase
SOD	superoxide dismutase
HO-1	heme oxygenase-1
Nrf2	nuclear factor erythroid 2-like 2
PPAR-γ	peroxisome proliferator-activated receptor gamma
BACE1	β-site-amyloid precursor protein cleaving enzyme 1; β-secretase 1
GSK3β	glycogen synthase kinase 3β
38MAPK	p38 mitogen-activated protein kinase
PGE2	prostaglandin E2
PI3K	phosphoinositide 3-kinase
AKT	protein kinase B, PKB, serine/threonine-specific protein kinases
MAO	monoamine oxidase A or B
GSH	glutathione
GSH-Px	glutathione peroxidase
TGF-β	transforming growth factor β
STAT1	signal transducer and activator of transcription 1
AChE	acetylcholine esterase
ChAT	choline acetyltransferase
AT1R	angiotensin II type 1 receptor
i.p.	intraperitoneal
i.g.	intragastric
